# 在线固相萃取/净化-高效液相色谱-串联质谱法同时测定番茄、大米和圆白菜中8种氨基甲酸酯类农药残留

**DOI:** 10.3724/SP.J.1123.2021.01028

**Published:** 2021-12-08

**Authors:** Xin LIU, Xiulan SUN, Jin CAO

**Affiliations:** 1.江南大学食品学院, 江苏 无锡 214122; 1. School of Food Science and Technology, Jiangnan University, Wuxi 214122, China; 2.中国食品药品检定研究院, 北京 100050; 2. National Institutes for Food and Drug Control, Beijing 100050, China

**Keywords:** 高效液相色谱-串联质谱, 在线固相萃取, 氨基甲酸酯类农药残留, 植物性食品, high performance liquid chromatography-tandem mass spectrometry (HPLC-MS/MS), online solid phase extraction, carbamate pesticide residues, plant foods

## Abstract

建立了在线固相萃取/净化-高效液相色谱-串联质谱(online SPE-HPLC-MS/MS)同时测定番茄、大米和圆白菜中8种氨基甲酸酯类农药的分析方法。将番茄5.0 g(不加水)、圆白菜和大米各2.0 g(各加3 mL水),以1000 r/min旋涡1 min,加入2 g氯化钠和10 mL 0.5%(v/v)甲酸乙腈溶液,旋涡均匀后离心,上清液氮吹后用10%(v/v)乙腈水溶液复溶,复溶液使用CAPCELL PAK C_18_净化柱(50 mm×2.0 mm, 15 μm)进行在线净化,当流动相0.1%(v/v)甲酸水溶液和乙腈的体积比分别为90:10和35:65时,可实现氨基甲酸酯农药的吸附和洗脱。以ACQUITY UPLC CSH C_18_色谱柱(100 mm×2.1 mm, 1.7 μm)为分析柱,实现8种氨基甲酸酯类农药的分离,以0.1%(v/v)甲酸水溶液和乙腈为流动相进行梯度洗脱,在12.0 min内即可完成分析。在优化条件下,8种氨基甲酸酯类农药在各自线性范围内线性良好,相关系数均大于0.995, LOD和LOQ分别为0.01~0.3 ng/mL和0.05~1.0 ng/mL;在3个加标水平下,8种氨基甲酸酯类农药的加标回收率为73.76%~112.32%,相对标准偏差为1.28%~13.14%(*n*=6)。通过在线净化的方式,大大提高了前处理效率,只需12 min即可完成净化上样,不需氮吹复溶等步骤,提高了处理效率。该法回收率高,重复性好,具有准确、快速、灵敏、环保等优点,可用于植物性食品中8种氨基甲酸酯类农药的检测。

## Abstract (en)

Carbamate pesticides are a class of synthetic pesticides having wide antimicrobial spectrum, good insecticidal efficacy, and a short residual period. These pesticides are used in agriculture, forestry, and animal husbandry. Their widespread use in the last two decades has led to the existence of drug residues in the environment, which are transferred to food, thereby raising concerns regarding the potential threat to human health. Rapid and accurate detection of carbamate pesticide residues in food is of great significance for food safety, and this requires pretreatment to purify the target components and maximize the accuracy and precision of the analysis. A rapid and accurate analytical method based on online solid phase extraction/purification-high performance liquid chromatography-tandem mass spectrometry (online SPE-HPLC-MS/MS) was established for the determination of eight carbamate pesticides in tomato, rice, and cabbage. About 5.0 g of tomato (without water), 2.0 g of cabbage, and 2.0 g of rice (mixed with 3 mL of water) were vortexed at 1000 r/min for 1 min. After adding 2 g of sodium chloride and 10 mL of acetonitrile containing 0.5% (v/v) formic acid, the samples were extracted and centrifuged. The supernatants were combined after the samples were extracted again. The reconstituted solutions were then purified on a CAPCELL PAK C_18_ column (50 mm×2.0 mm, 15 μm). When the volume ratio of 0.1% (v/v) formic acid aqueous solution and acetonitrile (used as the mobile phases) was 90:10 and 35:65, the eight carbamate pesticides could be completely adsorbed and eluted. The carbamate pesticides were separated on an ACQUITY UPLC CSH C_18_ column (100 mm×2.1 mm, 1.7 μm) under gradient elution and analyzed in the multiple reaction monitoring (MRM) mode with positive electrospray ionization (ESI^+^). Under the optimum conditions, the calibration curves of the eight carbamate pesticide residues showed good linearity (*r*>0.995) within their respective linear ranges. The limits of quantification (LOQs) and limits of detection (LODs) were in the range of 0.05-1.0 ng/mL (*S/N*=10) and 0.01-0.3 ng/mL (*S/N*=3). The recoveries were in the range of 73.76%-112.32% at three spiked levels (2, 10, and 20 ng/mL), with relative standard deviations of 1.28%-13.14% (*n*=6). The online purification method showed better enrichment and purification ability for the target substances than did the offline purification method and greatly improved the pretreatment efficiency. The loading and purification could be completed within 12 min. The developed method has the advantages of high recovery rate, good reproducibility, accuracy, rapidness, sensitivity, and environment friendliness. It can be used for the determination of the eight carbamate pesticides in plant foods, such as tomato, rice, and cabbage.

氨基甲酸酯类农药是一类分子中含有氨基甲酸酯结构的人工合成农药,是一种新型广谱的杀虫、杀螨除草剂^[1,2]^。自20世纪70年代以来,随着有机磷农药相继被不同国家禁用或者限制使用,氨基甲酸酯类农药因选择性强、药效好、残留期短且种类多,使用量逐年增加,被广泛应用于农业和林牧业中^[3,4]^。氨基甲酸酯类农药的广泛使用会导致其在食品中造成残留,从而威胁人类健康^[5,6]^。因此食品中氨基甲酸酯类农药残留的快速准确检测对保障食品安全具有重大意义,为减少复杂基质对检测结果的影响,需要样品前处理过程中能够最大限度地净化目标成分,除去目标杂质。

氨基甲酸酯类农药种类多,极性强,热稳定性差,气相色谱法不适用于测定非挥发性或热不稳定性农药^[7,8]^,因此目前大多采用灵敏度高、适用范围广的高效液相色谱-串联质谱技术进行测定^[9,10]^。在线固相萃取是区别于传统离线固相萃取的全自动在线净化方式,采用阀切换即可完成样品的上样、富集和纯化,溶剂使用量少,提高了检测的灵敏度和准确度^[11,12,13]^。堵燕钰等^[14]^和张海超^[15]^使用在线净化-液相色谱-高分辨质谱联用技术测定了食品中氨基甲酸酯类农药残留,方法的回收率稳定,重复性良好,精密度高,证明在线净化方法可满足氨基甲酸酯类农药残留检测的要求。

大米是人们日常生活饮食中重要的主食之一,番茄和圆白菜价格实惠,也是人们经常选择的蔬菜。我国国家标准^[16]^《食品安全国家标准食品中农药最大残留限量》中规定了氨基甲酸酯类农药在食品中的最大残留限量,其中灭多威(methomyl)在番茄和圆白菜中的最大残留限量均为0.2 mg/kg,甲萘威(carbaryl)在大米、番茄和圆白菜中的最大残留限量均为1 mg/kg,异丙威(isoprocarb)在大米中的最大残留限量为0.2 mg/kg,抗蚜威(pirimicarb)在番茄和圆白菜中的最大残留限量为0.5 mg/kg和5 mg/kg。严格的最大残留限量对样品前处理方法提出了更大的挑战,因此建立操作简单、准确高效的前处理方法具有重要意义^[17,18]^。

本文以常见的大米、番茄和圆白菜作为代表性基质,使用自行搭建的在线前处理/净化技术对目标物进行富集和纯化,结合高效液相色谱-串联质谱技术测定灭多威、抗蚜威、速灭威(metolcarb)、残杀威(propoxur)、甲萘威、异丙威、甲硫威(methiocarb)和仲丁威(fenobucarb)等8种氨基甲酸酯类农药。相对于以往多种氨基甲酸酯类农药分析中常用的离线固相萃取净化方法,该方法样品净化自动化程度高,简单快速,分析效率高,溶剂消耗少,成本低,可满足世界各国对氨基甲酸酯类农药残留限量的检测要求。

## 1 实验部分

### 1.1 仪器、试剂与材料

高效液相色谱-串联质谱仪(Qtrap 6500,美国AB Sciex公司);全自动智能氮吹仪(FV4,得泰仪器科技有限公司);旋涡混匀器(VORTEX-5,南京贝登医疗股份有限公司);医用离心机(GTR1-B,北京新时代北利医疗器械有限公司);机械超声波清洗机(北京世纪科玺公司);分析天平(XP205和AL204,德国Mettler公司); Milli-Q超纯水器(美国Millipore公司); ACQUITY UPLC CSH C_18_色谱柱(100 mm×2.1 mm, 1.7 μm,美国Waters公司); CAPCELL PAK C_18_净化柱(50 mm×2.0 mm, 15 μm,北京迪泽尔科技有限公司)。

灭多威、抗蚜威、速灭威、残杀威、甲萘威、异丙威、甲硫威、仲丁威标准品(纯度≥97.63%)均购自德国Dr. Ehrenstorfer公司。甲醇、乙酸乙酯、丙酮、乙腈、二氯甲烷(色谱纯),以及甲酸(质谱纯)购自美国Thermo Fisher Scientific公司;氯化钠(分析纯)购自国药集团化学试剂有限公司;实验用水为超纯水(电阻率≥18.2 MΩ·cm), Milli-Q Plus超纯水设备制得;大米、番茄和圆白菜均为实验室日常抽检样品。

### 1.2 标准溶液配制

分别准确称取8种氨基甲酸酯类标准品适量,用甲醇分别配制成1.0 mg/mL的标准储备溶液,于-20 ℃避光保存。取8种标准储备液各100 μL,用甲醇定容至10 mL,得到10 μg/mL的混合标准中间溶液,于4 ℃避光保存。使用10%(v/v)乙腈水溶液将10 μg/mL的混合标准中间液稀释为适当浓度的系列混合标准工作液。

### 1.3 样品提取

将番茄样品绞碎匀浆,圆白菜样品切碎匀浆,大米样品直接粉碎过40目筛。称取上述番茄5.0 g、圆白菜和大米各2.0 g(精确到0.1 g),置于50 mL聚丙烯离心管中,圆白菜和大米中加入3 mL水(番茄中不加水),以1000 r/min旋涡1 min,加入2 g氯化钠和10 mL 0.5%(v/v)甲酸乙腈溶液,于旋涡混合器上混合3 min,以5000 r/min离心5 min,将上清液转移至另一根离心管中,再用5 mL 0.5%(v/v)甲酸乙腈溶液重复以上提取过程,合并提取液。于40 ℃下氮吹至干,加入2 mL 10%(v/v)乙腈水溶液,涡旋溶解残渣后,过0.45 μm微孔滤膜。

### 1.4 样品在线净化

使用CAPCELL PAK C_18_柱作为在线净化柱,通过改变0.1%(v/v)甲酸水(A相)和乙腈(B相)的比例实现上样、淋洗、洗脱和柱平衡4个阶段(见表1)。在线净化系统流路图见图1,六通阀处于位置A(position A)时进行上样,使用注射器进样,使样品提取液充满1 mL定量环,同时使用初始流动相(A相和B相的体积比为9∶1)对在线固相萃取柱进行平衡。进样处理时将六通阀由position A切换至position B,先由初始流动相带动样品提取液流经在线固相萃取柱,使目标化合物完全吸附到在线固相萃取柱上,杂质流进废液,随后通过调节流动相的比例,使目标化合物从在线固相萃取柱上洗脱,此时收集洗脱液,上机测定。

**表1:** 在线固相萃取/净化的梯度条件

Step	Time/min	φ(A)/%	φ(B)/%	Position	Function
1	0.5	90	10	A	injection
2	0.5	90	10	B	loading
3	3.0	90	10	B	rinse
4	2.0	35	65	B	elution
5	2.0	10	90	B	elution
6	2.0	90	10	A	balance

A: 0.1%(v/v) formic acid aqueous solution; B: ACN.

**图 1:**
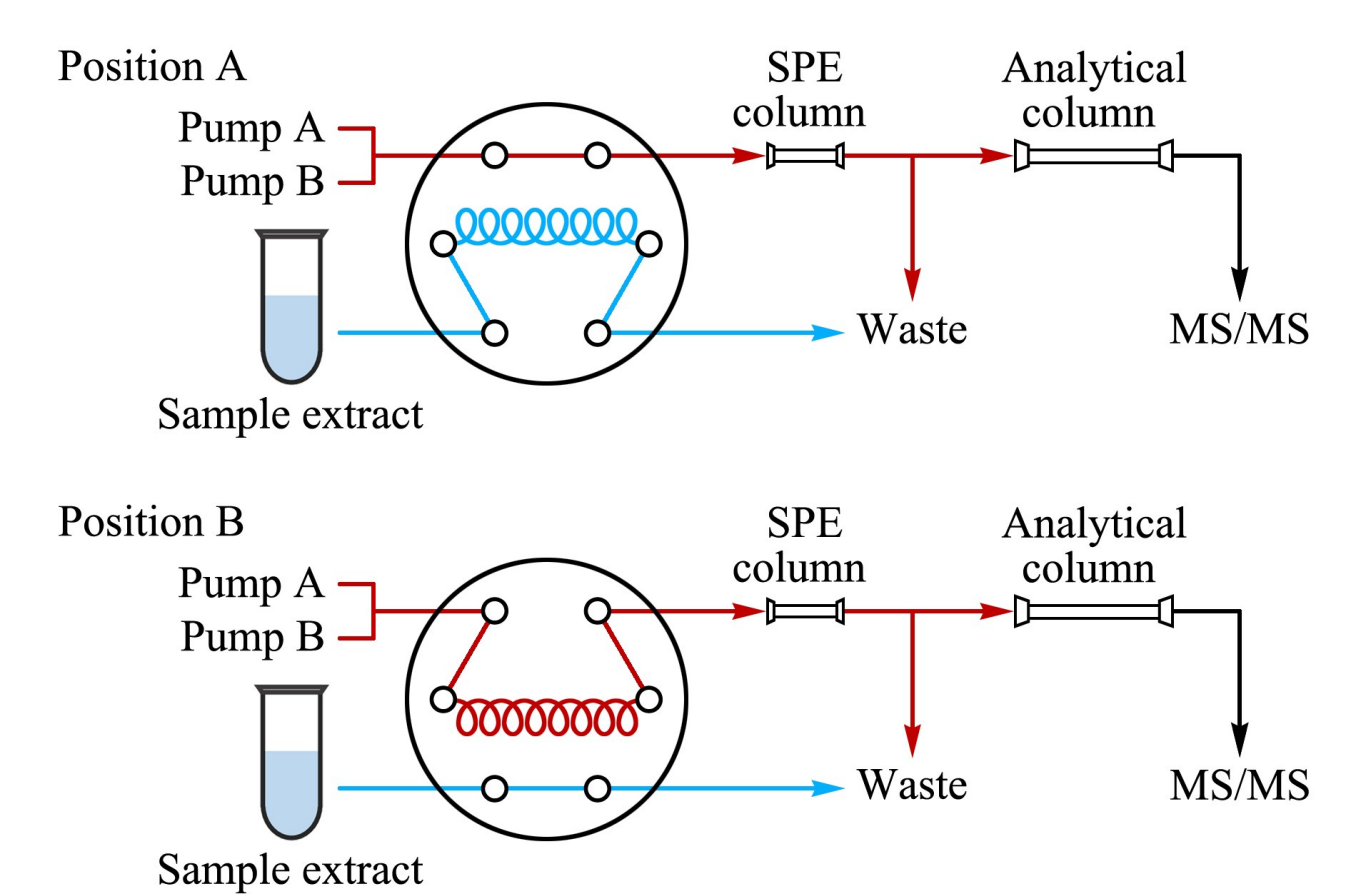
在线净化系统流路图

### 1.5 LC-MS/MS分析

1.5.1 色谱条件

分析柱为ACQUITY UPLC CSH C_18_色谱柱(100 mm×2.1 mm, 1.7 μm);柱温为40 ℃;流动相A为0.1%(v/v)甲酸水溶液,B为乙腈;流速为0.3 mL/min。梯度洗脱程序:0~1.0 min, 10%B; 1.0~4.0 min, 10%B~50%B; 4.0~5.0 min, 50%B~90%B; 5.0~8.0 min, 90%B; 8.0~8.1 min, 90%B~10%B; 8.1~10.0 min, 10%B。进样量为5 μL。

1.5.2 质谱条件

电离方式:电喷雾电离、正离子模式(ESI^+^);离子源温度:550 ℃;监测方式:多反应监测(MRM)模式;电喷雾电压(IS): 5.5 kV;雾化气压力(GS1): 344.75 kPa;辅助加热气压力(GS2): 344.75 kPa;气帘气压力(CUR): 275.8 kPa。8种氨基甲酸酯农药的保留时间、定性离子、定量离子、去簇电压和碰撞能见表2。

**表2:** 8种氨基甲酸酯类农药的保留时间和质谱参数

Compound	Retention time/min	Parent ion (m/z)	Daughter ion (m/z)	DP/ V	CE/ eV
Methomyl	3.78	163.1	106.1	85	18
			88.1^*^	85	18
Pirimicarb	3.90	239.1	182.2^*^	85	20
			72.2	85	20
Metolcarb	5.74	166.1	109.1^*^	85	15
			94.0	85	40
Propoxur	5.98	210.3	168.1	90	8
			111.2^*^	80	22
Carbaryl	6.14	202.2	145.1^*^	85	25
			127.1	85	38
Isoprocarb	6.33	194.3	137.2	50	15
			95.2^*^	85	20
Methiocarb	6.54	226.1	169.2	80	15
			121.2^*^	80	25
Fenobucarb	6.55	208.1	152.1	80	8
			95.0^*^	85	25

DP: declustering potential; CE: collision energy; * quantitative ion.

## 2 结果与讨论

### 2.1 色谱条件优化

选用填料粒径为1.7 μm的ACQUITY UPLC CSH C_18_色谱柱,其基于亚乙基桥颗粒基质的键合相,可以为物质的分离提供良好的峰形。实验考察了水-乙腈、0.1%甲酸水溶液-乙腈、0.1%甲酸水溶液-甲醇等流动相对色谱分离及灵敏度的影响。发现使用乙腈作为有机相时,多数物质的响应要好于甲醇。同时,在正离子模式下,水相中加入甲酸,有助于分析物形成[M+H]^+^,峰形更对称,也提高了分析灵敏度,因此本实验最终使用0.1%甲酸水溶液-乙腈作为流动相。通过优化梯度洗脱程序,8种氨基甲酸酯农药在10 min内即可达到最佳分离,最佳条件下的色谱图见图2。

**图2:**
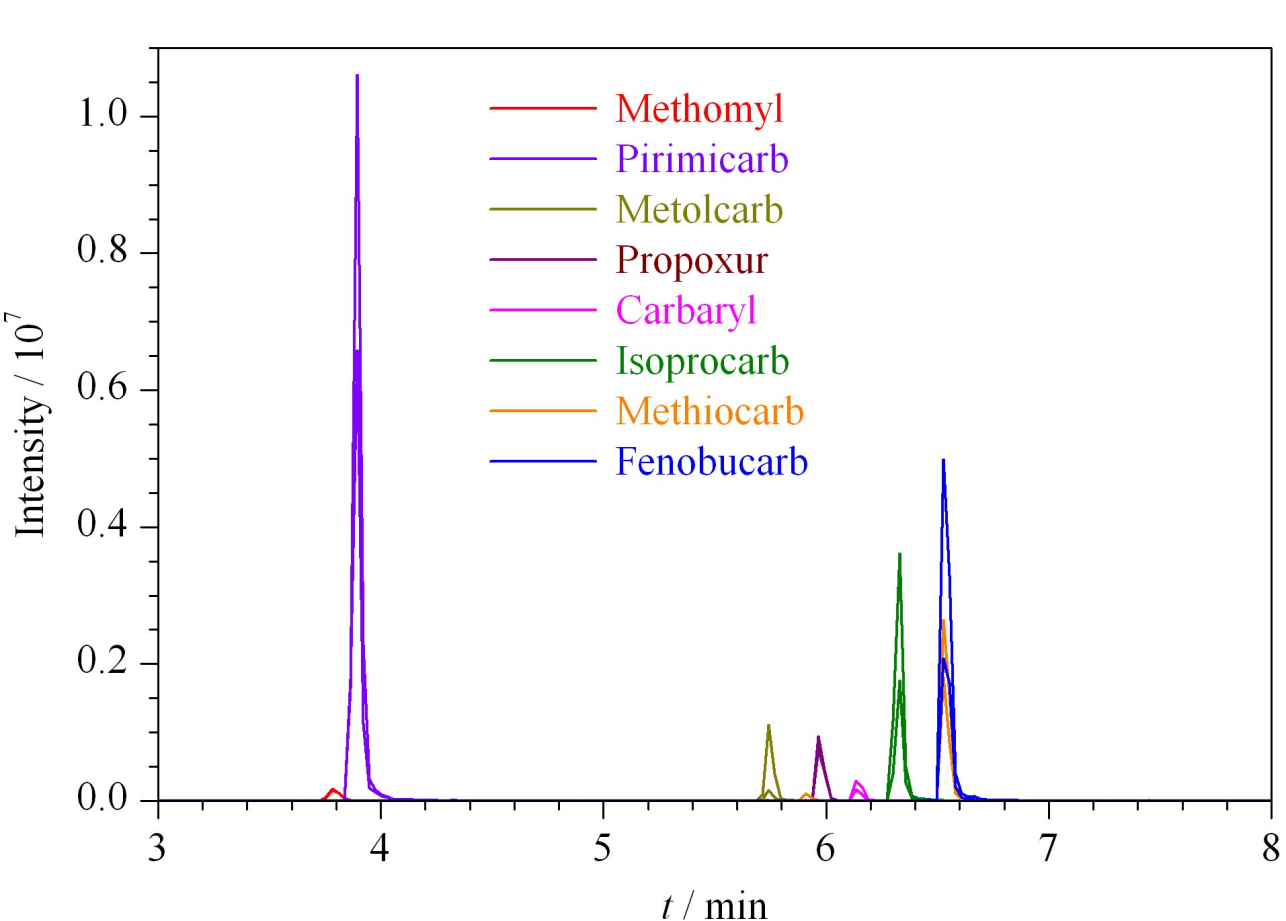
8种氨基甲酸酯农药混合标准溶液(100 ng/mL)的提取离子流色谱图

### 2.2 质谱条件优化

根据氨基甲酸酯类农药分子结构的特征,选择了电喷雾电离、正离子模式,用注射泵分别将质量浓度为100 ng/mL的单标准溶液以7 μL/min的流速注入质谱仪中,首先进行一级质谱分析,找到每种药物稳定的母离子[M+H]^+^峰。然后分别以8种药物的分子离子作为母离子进行子离子扫描分析,通过调节碰撞能,选取各目标物质的最优子离子。以信号最强的子离子作为定量离子,信号次强的子离子作为定性离子,对各离子对进行去簇电压、碰撞能、碰撞室入口电压及出口电压的优化,确定各目标物质的质谱参数(见表1),确保每种物质的响应和离子化效率达到最佳。

### 2.3 样品提取溶剂的选择

根据待测样品及目标物的性质进行提取溶剂的选择。本实验采用甲醇、乙酸乙酯、丙酮、乙腈和0.5%(v/v)甲酸乙腈溶液作为提取溶剂,进行加标回收试验来比较提取效果,加标水平为20 μg/kg,每个水平平行测定6次(见图3)。

**图3:**
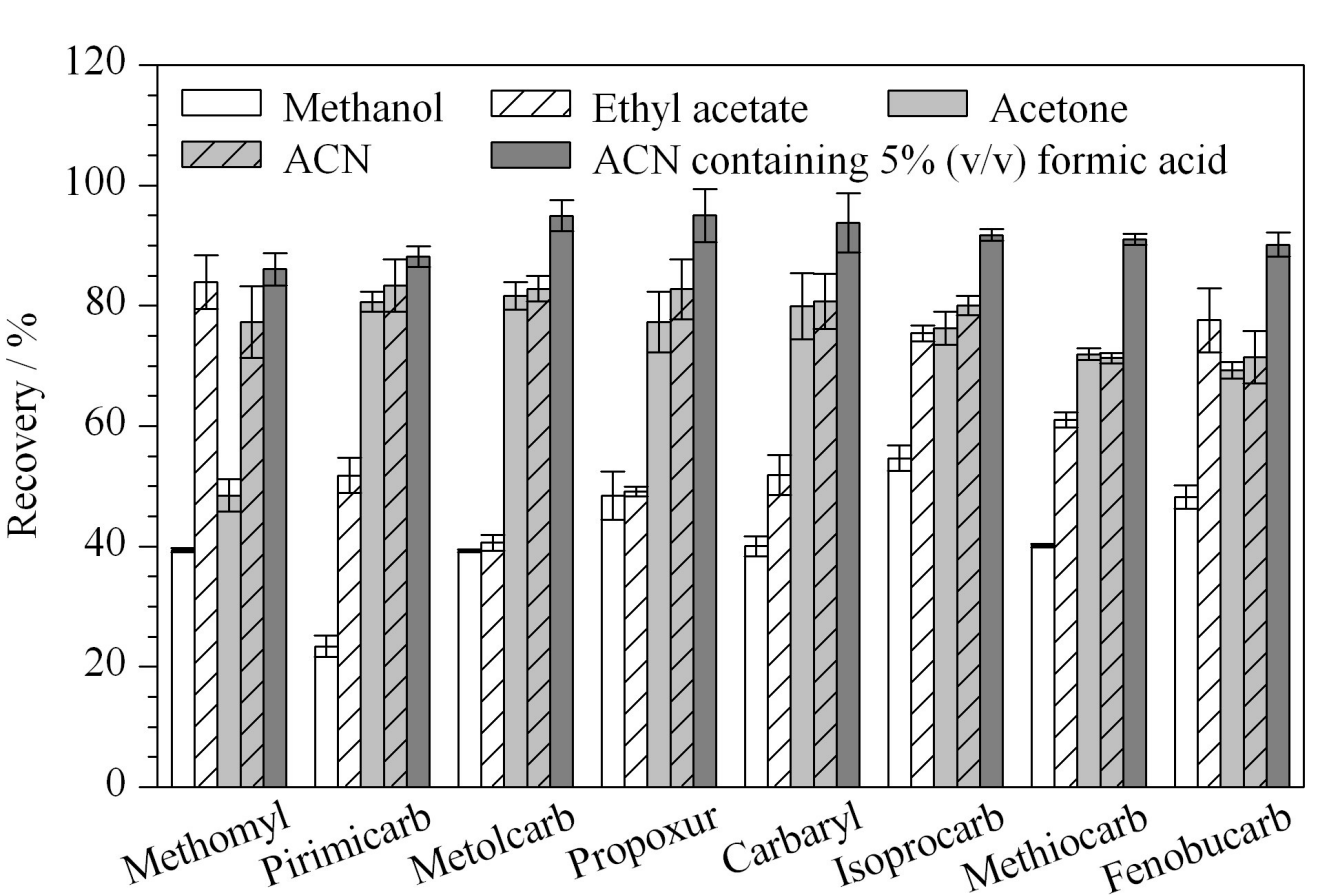
不同提取溶剂对8种氨基甲酸酯类农药回收率的影响(*n*=6)

结果发现,使用丙酮和乙酸乙酯提取时,番茄样品溶出的色素较多,使用甲醇作为提取溶剂时回收率在50%以下。使用0.5%甲酸乙腈溶液提取时回收率最高,这是因为乙腈通用性和组织穿透能力强,而且甲酸提供了酸性环境,有利于氨基甲酸酯类农药的稳定,故选用0.5%甲酸乙腈作为提取溶剂。

### 2.4 复溶溶剂中乙腈体积分数的优化

样品提取液氮吹后使用乙腈水溶液作为复溶溶剂。复溶溶剂中乙腈的体积分数会影响净化柱对目标化合物的吸附。乙腈体积分数越小,目标化合物越稳定吸附富集到前处理柱上。实验对比了乙腈体积分数分别为80%、60%、40%、30%、20%、10%时8种氨基甲酸酯类农药的回收率,结果见图4。当乙腈体积分数不高于10%时,8种氨基甲酸酯类农药均可完全富集到前处理柱上,实验最终确定复溶溶剂中乙腈的体积分数为10%。

**图4:**
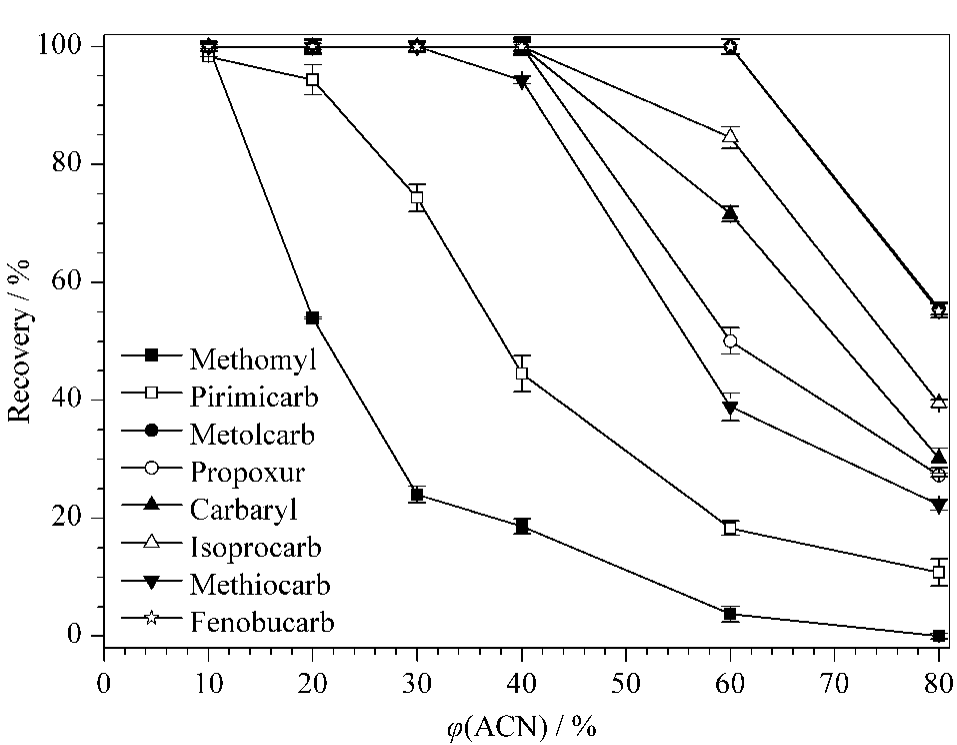
复溶溶剂中乙腈的体积分数对8种氨基甲酸酯农药回收率的影响(*n*=6)

### 2.5 在线固相萃取条件的优化

在线固相萃取包括上样、淋洗、洗脱和平衡4个步骤。本实验使用CAPCELL PAK C_18_柱作为在线净化柱,其填充剂为无定形硅胶键合十八烷基,可用于大部分在反相体系中进行分离的物质的富集纯化。本实验对流动相的种类及流速、上样和洗脱时流动相比例进行了优化。

2.5.1 流动相

实验对比分析了水-乙腈和0.1%(v/v)甲酸水溶液-乙腈作为流动相时目标分析物的富集纯化效果。发现当使用纯水作为水相时,目标物在上样阶段无法吸附到在线净化柱上,使用0.1%(v/v)甲酸水溶液作为水相时,可以满足目标物在净化柱上有效的富集和洗脱。

2.5.2 流动相比例

上样时,流动相中的乙腈体积分数越小,净化柱对8种氨基甲酸酯类农药的富集作用越强。灭多威的回收率受流动相中乙腈体积分数的影响较大,当乙腈体积分数高于35%时,灭多威的回收率仅为5%;当乙腈体积分数不高于10%时,灭多威的回收率才可达到95%以上。当流动相中乙腈体积分数低于30%时,其他7种氨基甲酸酯类农药即可完全吸附到净化柱上,故确定上样时流动相中乙腈的体积分数为10%。

洗脱时,当流动相中乙腈体积分数低于55%时,仲丁威和甲硫威均不能被洗脱;当乙腈体积分数为65%及以上时,所有的物质均可被完全洗脱,经色谱分析,洗脱液中乙腈的体积分数对色谱峰形及灵敏度无影响,均能保持尖锐对称的峰形,故确定洗脱时流动相中乙腈的体积分数为65%。

### 2.6 方法学考察

2.6.1 线性关系和检出限

配制每种化合物的系列混合标准溶液,按确定的分析条件进行测定,以各物质的质量浓度为横坐标、定量离子峰面积为纵坐标进行线性回归,绘制标准工作曲线。结果表明,各物质在各自线性范围内线性关系良好,线性相关系数(*r*)均大于0.995(见表3)。

**表3:** 8种氨基甲酸酯农药的线性方程、线性范围、相关系数、检出限和定量限

Compound	Linear equation	Linear range/(ng/mL)	r	LOD/(ng/mL)	LOQ/(ng/mL)
Methomyl	y=1.08×10^4^x+2.51×10^3^	0.5-50	0.9953	0.05	0.15
Pirimicarb	y=3.64×10^5^x+5.03×10^3^	0.1-100	0.9951	0.01	0.05
Metolcarb	y=3.63×10^4^x+4.15×10^2^	0.1-100	0.9991	0.04	0.10
Propoxur	y=2.60×10^4^x+4.76×10^3^	0.5-100	0.9970	0.05	0.15
Carbaryl	y=9.40×10^3^x+1.87×10^3^	1.0-100	0.9992	0.30	1.0
Isoprocarb	y=1.17×10^5^x+1.11×10^4^	0.1-100	0.9984	0.03	0.10
Methiocarb	y=9.29×10^4^x+3.03×10^3^	0.5-100	0.9970	0.05	0.20
Fenobucarb	y=1.74×10^5^x+1.64×10^4^	0.5-100	0.9979	0.06	0.20

y: peak area; x: mass concentration, ng/mL.

以各目标化合物信噪比(*S/N*)分别为3和10时计算检出限(LOD)和定量限(LOQ), 8种氨基甲酸酯类农药的LOD和LOQ分别为0.01~0.30 ng/mL和0.05~1.0 ng/mL,均较低于文献值。堵燕钰等^[14]^对茶叶中5种氨基甲酸酯类农药进行检测的检出限为0.3~1 μg/kg,张海超^[15]^对5种蔬菜基质中24种氨基甲酸酯类农药进行检测的定量限为1 μg/kg和5 μg/kg。

2.6.2 回收率和精密度

对阴性番茄、大米和圆白菜基质进行3个水平的加标回收试验,使得最终净化液中各物质的加标水平分别为2、10、20 ng/mL,每个水平进行6组平行实验,计算回收率及相对标准偏差(见表4)。结果表明,8种氨基甲酸酯类农药在3个加标水平下的加标回收率为73.76%~112.32%,相对标准偏差为1.28%~13.14%,说明方法具有良好的准确性和精密度。

**表4:** 8种氨基甲酸酯类农药在3种基质中的回收率和相对标准偏差(n=6)

Compound	Spiked level/ (ng/mL)	Tomato			Rice			Cabbage	
		Recovery/%	RSD/%		Recovery/%	RSD/%		Recovery/%	RSD/%
Methomyl	2	80.40	5.88		106.81	3.58		108.59	5.11
	10	86.07	5.77		94.41	8.92		93.79	11.92
	20	75.98	8.65		85.87	10.55		75.32	6.51
Pirimicarb	2	80.03	4.37		102.35	1.79		107.36	8.59
	10	84.36	2.42		97.79	7.82		100.06	4.34
	20	83.19	4.47		111.76	4.67		88.67	6.18
Metolcarb	2	95.84	3.89		92.38	1.28		101.92	2.27
	10	94.50	2.31		101.28	11.95		95.33	2.64
	20	88.50	3.51		97.35	2.96		85.97	3.56
Propoxur	2	77.37	9.99		82.49	4.56		101.33	8.19
	10	78.89	5.60		96.52	7.05		94.95	6.06
	20	111.62	4.98		90.41	7.26		84.01	13.14
Carbaryl	2	74.98	8.13		87.72	7.00		86.09	5.19
	10	82.35	3.84		94.04	6.80		89.69	9.74
	20	73.76	9.05		92.17	3.21		81.22	7.60
Isoprocarb	2	112.32	3.94		86.96	3.13		105.35	3.90
	10	103.45	1.41		82.35	4.59		109.01	4.73
	20	92.81	2.54		89.34	2.92		101.06	3.11
Methiocarb	2	82.65	4.36		82.77	3.24		97.41	2.45
	10	84.61	1.92		91.08	7.81		84.50	5.45
	20	75.35	3.51		82.61	4.68		93.92	5.50
Fenobucarb	2	94.84	2.94		100.63	1.91		101.28	3.57
	10	97.10	2.24		103.01	2.99		98.58	5.23
	20	91.03	3.71		95.72	2.71		97.01	4.65

2.6.3 在线净化柱的重复性

使用CAPCELL PAK C_18_柱进行200次实验后,使用混合标准溶液上样并收集洗脱液,与混合溶液同时进行质谱分析与定量,发现洗脱液与原混合溶液中8种氨基甲酸酯类农药的含量相差在5%以内,说明净化柱的效能稳定,重复使用性强。

### 2.7 与离线净化方法的比较

采用现行标准NY/T 1679-2009《植物性食品中氨基甲酸酯类农药残留的测定 液相色谱-串联质谱法》的离线固相萃取净化方法时,番茄、大米和圆白菜中8种氨基甲酸酯类农药的加标回收率为55.68%~99.13%, RSD为1.48%~26.46%,表明离线方法的重复性和准确度均不如在线净化方法。

在实验操作上,离线SPE净化方法的整个净化过程近80 min,而在线净化方法无需进行上样洗脱等人工操作及氮气吹干,整个净化过程仅需12 min,有效简化了样品的前处理步骤,也缩短了前处理的时间,减少了有机溶剂的和耗材的使用量。

### 2.8 实际样品的测定

根据优化的净化条件与仪器条件,对实验室的番茄、大米和圆白菜各10份样品进行检测。结果表明,抽检的30份样品中均未检出氨基甲酸酯类农药。说明此次抽检的样品总体质量较好。在加标回收率实验中已经证明本方法的回收率高,重复性好,因此可以对样品中的氨基甲酸酯类农药进行有效监测。

## 3 结论

本文采用在线固相萃取,结合高效液相色谱-串联质谱法对番茄、大米和圆白菜中8种氨基甲酸酯类农药进行富集纯化和分析检测。该法操作简单,准确度和回收率高,重复性好,具有准确、快速、灵敏、环保等优点,可满足番茄、大米和圆白菜中氨基甲酸酯类农药分析的要求。与离线固相萃取方法相比,该方法在准确度、灵敏度和重复性,以及前处理操作步骤和所需时间等方面都具有明显的优势。

农药种类繁多,性质也千差万别,可继续开发针对其他农药的在线净化-高效液相色谱-串联质谱法,从而为我国食品中农药残留的监测提供技术支持。在此基础上,可开发更多种类的填料应用到在线净化柱中,并将不同填料的净化柱的净化效果进行对比,为日后研究提供参考。
